# MiR-26a functions oppositely in osteogenic differentiation of BMSCs and ADSCs depending on distinct activation and roles of Wnt and BMP signaling pathway

**DOI:** 10.1038/cddis.2015.221

**Published:** 2015-08-06

**Authors:** X Su, L Liao, Y Shuai, H Jing, S Liu, H Zhou, Y Liu, Y Jin

**Affiliations:** 1 Department of Orthodontics, Stomatology Hospital of Xi'an Jiaotong University College of Medicine, Xi'an, Shaanxi 710004, China; 2 State Key Laboratory of Military Stomatology, Center for Tissue Engineering, School of Stomatology, Fourth Military Medical University, Xi'an, Shaanxi 710032, China; 3 Institute of Neurobiology, Environment and Genes Related to Diseases, Key Laboratory of Education Ministry, Xi'an Jiaotong University College of Medicine, Xi'an, Shaanxi 710061, China; 4 Research and Development Center for Tissue Engineering, Fourth Military Medical University, Xi'an, Xi'an, Shaanxi 710032, China; 5 State Key Laboratory of Military Stomatology, Department of Oral Histology and pathology, School of Stomatology, Fourth Military Medical University, Xi'an, Shaanxi 710032, China

## Abstract

MicroRNAs (miRNAs) emerge as important regulators of stem cell lineage commitment and bone development. MiRNA-26a (miR-26a) is one of the important miRNAs regulating osteogenic differentiation of both bone marrow-derived mesenchymal stem cells (BMSCs) and adipose tissue-derived mesenchymal stem cells (ADSCs). However, miR-26a functions oppositely in osteogenic differentiation of BMSCs and ADSCs, suggesting distinct post-transcriptional regulation of tissue-specific MSC differentiation. However, the molecular basis is largely unknown. Here, we report that the function of miR-26a is largely depended on the intrinsic signaling regulation network of MSCs. Using bioinformatics and functional assay, we confirmed that miR-26a potentially targeted on GSK3*β* and Smad1 to regulate Wnt and BMP signaling pathway. Overall comparative analysis revealed that Wnt signaling was enhanced more potently and played a more important role than BMP signaling in osteogenic differentiation of BMSCs, whereas BMP pathway was more essential for promoting osteogenic differentiation of ADSCs. The distinct activation pattern and role of signaling pathways determined that miR-26a majorly targeted on GSK3*β* to activate Wnt signaling for promoting osteogenic differentiation of BMSCs, whereas it inhibited Smad1 to suppress BMP signaling for interfering with the osteogenic differentiation of ADSCs. Taken together, our study demonstrated that BMSCs and ADSCs applied different signaling pathway to facilitate their osteogenic differentiation, which determined the inverse function of miR-26a. The distinct transcriptional regulation and post-transcriptional regulation network suggested the intrinsic molecular differences between tissue-specific MSCs and the complexity in MSC research and MSC-based cell therapy.

Our understanding of the molecular mechanisms governing differentiation of mesenchymal stem cells (MSCs) developed rapidly in the past decades. A number of secretory molecules and transcription factors have been identified as regulators controlling osteoblastogenesis.^1, 2, 3^ Secreted molecules including bone morphogenetic proteins (BMPs), Wnt proteins, Indian hedgehog (IHH) and fibroblast growth factors (FGFs) are necessary for osteoblast differentiation and bone development.^1, 3^ These secreted molecules activate different signaling pathways through autocrine or paracrine signaling to regulate the expression of a set of transcription factors. Osteoblast-specific (Runx2, Osterix and ATF4) and nonspecific factors, expressing at distinct time points during the differentiation process, determine the lineage commitment of MSCs.^1, 2^

One recent breakthrough is that microRNAs (miRNAs), a class of 22–24 bp noncoding RNAs, emerge as important regulatory mechanism of MSC lineage commitment and bone development.^4, 5^ Several miRNAs have been identified as important regulators of osteogenesis.^6^ Among them, miR-26a is one of the important miRNAs regulating the osteogenic differentiation of both bone marrow-derived MSCs (BMSCs) and adipose tissue-derived MSCs (ADSCs). Expression of miR-26a is significantly increased in both BMSCs and ADSCs under osteogenic induction.^7, 8, 9^ Our recent work confirmed that miR-26a is a promoter of BMSC osteogenic differentiation.^10^ However, Luzi *et al.*^11, 12^ showed that miR-26a suppresses the osteogenic differentiation of ADSCs. These contradictory observations indicated that miR-26a plays distinct post-transcriptional regulatory function between BMSCs and ADSCs. However, the molecular basis is totally unknown.

BMSCs, which are capable of self-renewal and multipotent differentiation, play important role in bone homeostasis and regeneration.^13^ Among MSCs isolated from several connective tissues, ADSCs sharing many biological characteristics with BMSCs are attractive alternatives for cell therapy.^14, 15, 16^ Both BMSCs and ADSCs could potently differentiate into osteoblasts under similar induction conditions^17^ and have been applied to bone repair and regeneration in clinic trials.^18, 19, 20^ However, notwithstanding the similarity of cell behavior and function *in vitro*, BMSCs and ADSCs have distinct origin, location and physiological function *in vivo*. This raises the question of whether the molecular basis of osteogenic differentiation between BMSCs and ADSCs is identical. Indeed, a number of recent studies based on profiling strategy showed differences at transcriptional and proteomic levels between BMSCs and ADSCs.^21, 22, 23, 24, 25, 26^ Importantly, a number of differentially expressed genes are involved in Wnt signaling and other differentiation pathways,^22, 26^ suggesting a difference in signaling regulation programs between BMSCs and ADSCs. However, no conclusive functional study confirms that BMSCs and ADSCs adopt different sets of gene regulation programs for osteogenic differentiation.

The opposing function of miR-26a further suggests that the regulation program of BMSC and ADSC differentiation is different at post-transcriptional level. As one miRNA targets on numbers of mRNAs,^27^ the opposing function of miR-26a might be due to it targeting on different osteogenic regulators in BMSCs and ADSCs. In this study, we showed that the function of miR-26a is largely depended on the intrinsic signaling regulation network of MSCs. This finding indicates that there are specific signaling regulation networks between different MSCs, suggesting the complexity of tissue-specific MSC research and application in bone regeneration.

## Results

### MiR-26a functions oppositely in osteogenic differentiation of BMSCs and ADSCs

**Figure 1:**
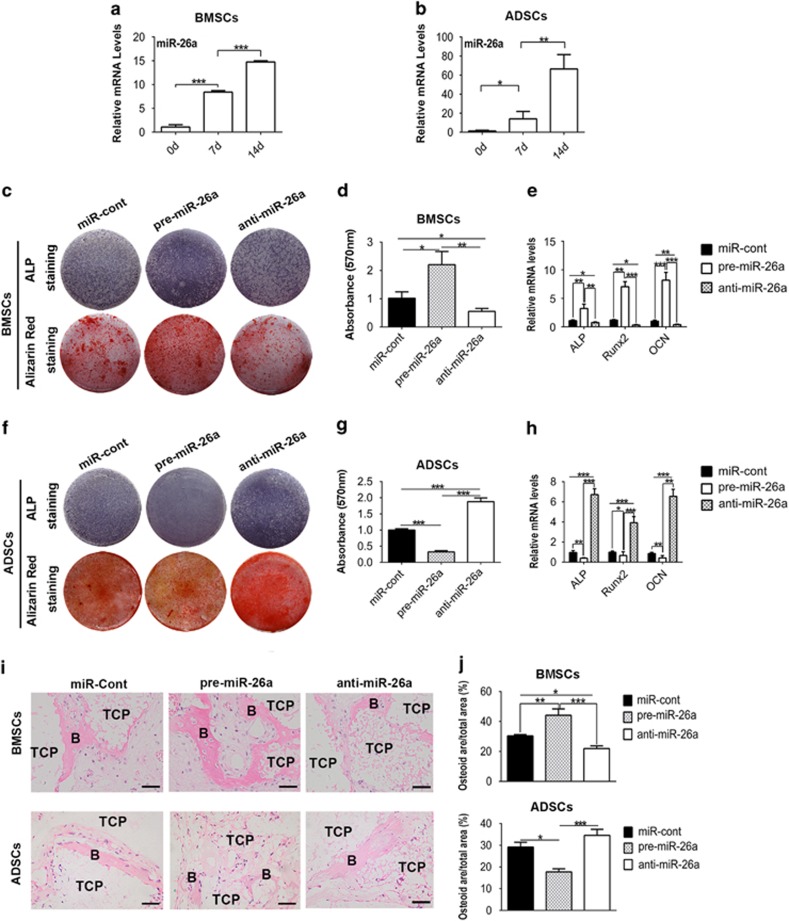
MiR-26a inversely affects the osteogenic differentiation of BMSCs and ADSCs. (**a** and **b**) Expression of miR-26a in BMSCs (**a**) and ADSCs (**b**) during osteogenic induction. **(c–h**) BMSCs (**c–e**) and ADSCs (**f–h**) were transfected with miR-26a precursors (pre-miR-26a), miR-26a inhibitors (anti-miR-26a) and negative control (miR-cont) for 48 h before osteogenic induction. ALP staining and alizarin red staining were performed after 7 days or 14 days of induction separately (**c** and **f**). Alizarin red staining was extracted with cetylpyridinium chloride and quantified by spectrophotometer (**d** and **g**). Expression of *Alp*, *Runx2* and *Ocn* (normalized to *β*-actin) was determined by real-time RT-PCR (**e** and **h**). (**i**) BMSCs and ADSCs transfected with miR-26a precursors, miR-26a inhibitors and negative control *in vitro* were transplanted with HA-TCP subcutaneously into immunocompromised mice for 8 weeks. The transplants were harvested and stained with H&E. B, bone; TCP, hydroxyaptite-tricalcium phosphate. (**j**) Osteoid formation in transplants was evaluated as osteoid area per total area in H&E staining photos with Image Pro software. Scale bar: 200 *μ*m. Results are shown as mean±S.D. *P<0.05, **P<0.01, ***P<0.001 (*n*=4)

To investigate the function of miR-26a in osteogenic differentiation of BMSCs and ADSCs, we cultured BMSCs and ADSCs in a medium containing ascorbic acid, *β*-glycerophosphate and dexamethasone, widely used in *in vitro* osteogenesis model.^28^ After osteogenic induction, BMSCs and ADSCs highly expressed *Alp* (alkaline phosphatase, a marker of early osteogenic differentiation) and *Ocn* (osteocalcin, a critical marker of mature osteoblasts), and formed mineralized nodules (Supplementary Figure S1D–G). Notably, the expression of miR-26a was significantly increased during osteogenic differentiation of both BMSCs and ADSCs (Figures 1a and b).

To investigate the role of miR-26a in BMSC and ADSC differentiation, we performed gain- and loss-of-function assay. Synthesized pre-miR-26a or anti-miR-26a was transfected to efficiently overexpress or knock down miR-26a separately (Supplementary Figure S2). In BMSCs, overexpression of miR-26a increased ALP activity, *Alp* mRNA and *Runx2* mRNA expression at day 7, and mineralized nodule formation and *Ocn* expression at day 14 (Figures 1c–e), mirroring what was seen in knockdown of miR-26a. In contrast, overexpression of miR-26a significantly inhibited osteogenic differentiation of ADSCs, whereas knockdown of miR-26a promoted ADSC osteogenic differentiation (Figures 1f–h).

To further confirm the function of miR-26a *in vivo*, we transplanted MSCs transfected with pre-miR-26a, anti-miR-26a or negative control subcutaneously into immunocompromised mice. H&E staining of implants showed that BMSC-overexpressed miR-26a (BMSC/pre-miR-26a) formed more bone tissue, whereas BMSC/anti-miR-26a formed less than control. Conversely, overexpression of miR-26a inhibited the *in vivo* bone formation of ADSCs, whereas knockdown of miR-26a promoted ADSC bone formation (Figures 1i and j). Consistent with previous studies,^10, 11^ our results indicated that miR-26 promotes BMSC osteogenic differentiation but inhibits ADSC osteogenic differentiation.

### MiR-26a targets on both GSK3*β* and Smad1

**Figure 2:**
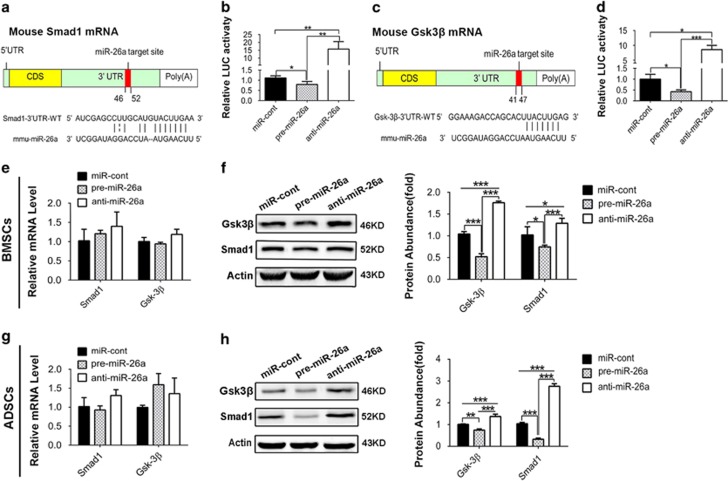
MiR-26a targets on both Smad1 and GSK3*β* mRNA. (**a**) The diagram showed the binding region of miR-26a to 3′ UTR of Smad1 by complementary base paring. (**b**) Luciferase activity of pMIR-Reporter containing Smad1 3′ UTR was measured 48 h after co-transfection with pre-miR-26a, anti-miR-26a or miR-control. (**c**) The diagram showed the binding region of miR-26a to 3′ UTR of GSK3*β*. (**d**) Luciferase activity of pMIR-Reporter containing GSK3*β* 3′ UTR was measured 48 h after co-transfection. (**e–h**) BMSCs and ADSCs were transfected with pre-miR-26a, anti-miR-26a or miR-control for 48 h. *Smad1* and *GSK3β* mRNA level in BMSCs (**e**) and ADSCs (**g**) was determined by real-time RT-PCR. Smad1 and GSK3*β* protein accumulation in BMSCs (**f**) and ADSCs (**h**) was determined by western blot. Relative protein abundance of each blots was normalized to the gray value of *β*-actin. Results are represented as mean±S.D. *P<0.05, **P<0.01, ***P<0.001 (*n*=3)

To investigate the molecular basis of miR-26a, we employed three miRNA target prediction databases (TargetScan (http://www.targetscan.org/mmu_50/), PicTar (http://pictar.mdc-berlin.de/) and TargetRank (http://genes.mit.edu/targetrank/)) to predict the target mRNA. Among the predicted mRNAs that could regulate osteogenesis, Smad1 (Figure 2a) and GSK3*β* (Figure 2c) have been experimentally certified.^11, 29, 30, 31^ To confirm the direct binding of miR-26a on Smad1 and GSK3*β* mRNAs, we performed luciferase activity assay by co-transfecting pMIR reporter containing the binding sites of Smad1 or GSK3*β* 3′ UTR with pre-miR-26a or anti-miR-26a. Confirmative with the *in silico* prediction, overexpression of miR-26a inhibited the luciferase activity of reporters of Smad1 and GSK3*β*, whereas knockdown of miR-26a increased the luciferase activity of both reporters (Figures 2b and d). Gain- and loss-of-function assays further confirmed that miR-26a decreased both Smad1 and GSK3*β* protein accumulation in BMSCs (Figure 2f) and ADSCs (Figure 2h). Real-time reverse transcription-PCR (RT-PCR) showed that the mRNA levels of *Smad1* and *GSK3β* were not significantly changed after overexpression or knockdown of miR-26a (Figures 2e and g), indicating that miR-26a functions through post-transcriptional regulation.

### MiR-26a regulates the osteogenic differentiation of BMSCs and ADSCs through different targets

**Figure 3:**
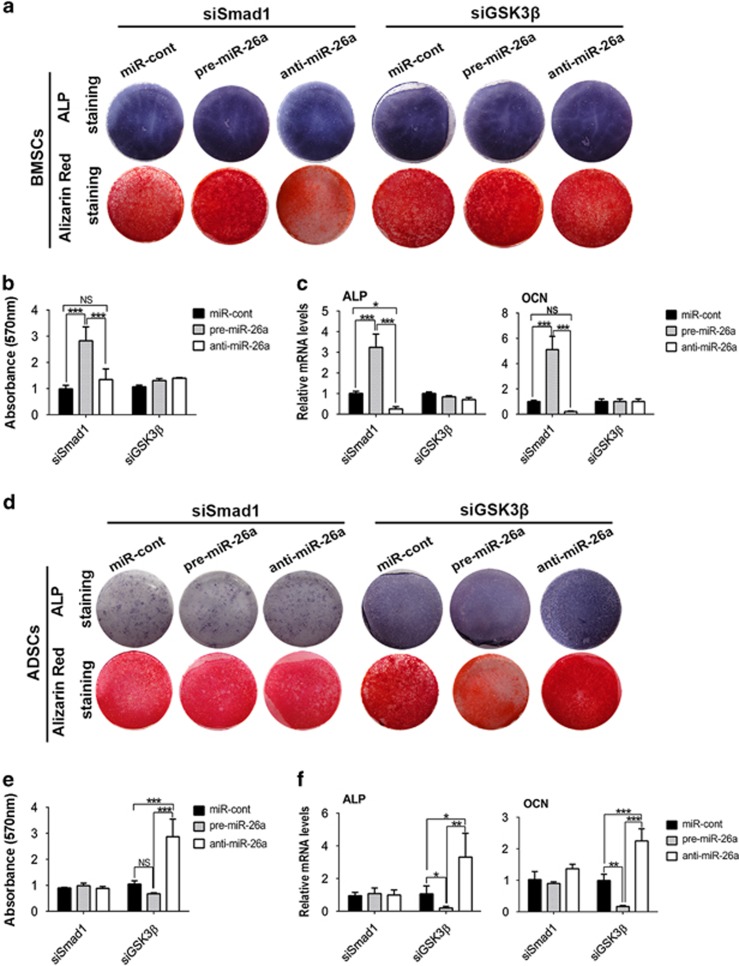
MiR-26a focuses on different targets to control BMSC and ADSC osteogenic differentiation. Smad1 or GSK3*β* siRNA was co-transfected with negative control, pre-miR-26a and anti-miR-26a into BMSCs (**a–c**) or ADSCs (**d–f**) for 48 h before osteogenic induction. (**a, b, d** and **e**) ALP staining and alizarin red staining were performed after osteogenic induction. (**c** and **f**) *Alp* and *Ocn* mRNA expression was analyzed by real-time RT-PCR after induction. Results are shown as mean±S.D. *P<0.05, **P<0.01, ***P<0.001 (*n*=3)

As Smad1 is the positive regulator but GSK3*β* is the negative regulator of BMSC osteogenesis,^32, 33^ we moved forward to explore which was the major target of miR-26a by knocking down GSK3*β* or Smad1 using specific siRNA (Supplementary Figure S3). After knocking down of GSK3*β*, miR-26a no longer obviously affected the osteogenic differentiation of BMSCs. However, miR-26a still potently promoted BMSC osteogenic differentiation after knocking down of Smad1 (Figures 3a–c). On the contrary, knockdown of Smad1 prohibited the function of miR-26a on osteogenic differentiation of ADSCs, but miR-26a still inhibited osteogenic differentiation of ADSCs after knocking down of GSK3*β* (Figures 3d–f). Taken together, these results indicated that miR-26a functions majorly by inhibiting GSK3*β* to promote osteogenesis of BMSCs, and restraining Smad1 to suppress ADSC differentiation.

### GSK3*β* and Smad1 have distinct importance in osteogenic differentiation of BMSCs and ADSCs

**Figure 4:**
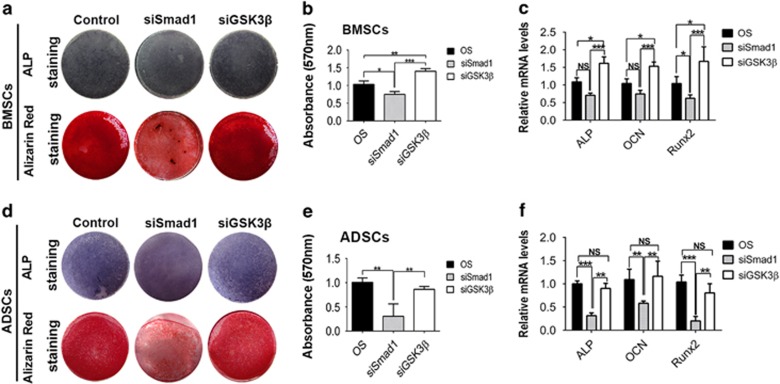
GSK3*β* and Smad1 have distinct importance in osteogenic differentiation of BMSCs and ADSCs. (**a, b, d** and **e**) ALP staining and alizarin red staining of BMSCs (**a** and **b**) and ADSCs (**d** and **e**) transfected with GSK3*β* siRNA, Smad1 siRNA or negative control after osteogenic induction. (**c** and **f**) *Alp*, *Ocn* and *Runx2* mRNA expression was determined by real-time RT-PCR. Results are shown as mean±S.D. *P<0.05, **P<0.01, ***P<0.001 (*n*=3)

The observation that miR-26a targeted on both GSK3*β* and Smad1 but functioned majorly by regulating different targets in BMSCs and ADSCs raised a question. One possible answer is that GSK3*β* and Smad1 have different importance in the regulation of BMSC and ADSC differentiation. To verify this hypothesis, we compared the function of GSK3*β* and Smad1 between BMSCs and ADSCs through loss-of-function assay. As expected, GSK3*β* siRNA significantly promoted osteogenic differentiation of BMSCs (Figures 4a–c), whereas it caused no significant changes in ADSCs (Figures 4d–f). Smad1 siRNA significantly decreased osteogenic differentiation of ADSCs (Figures 4d–f), but only slightly affected the differentiation of BMSCs (Figures 4a–c).

**Figure 5:**
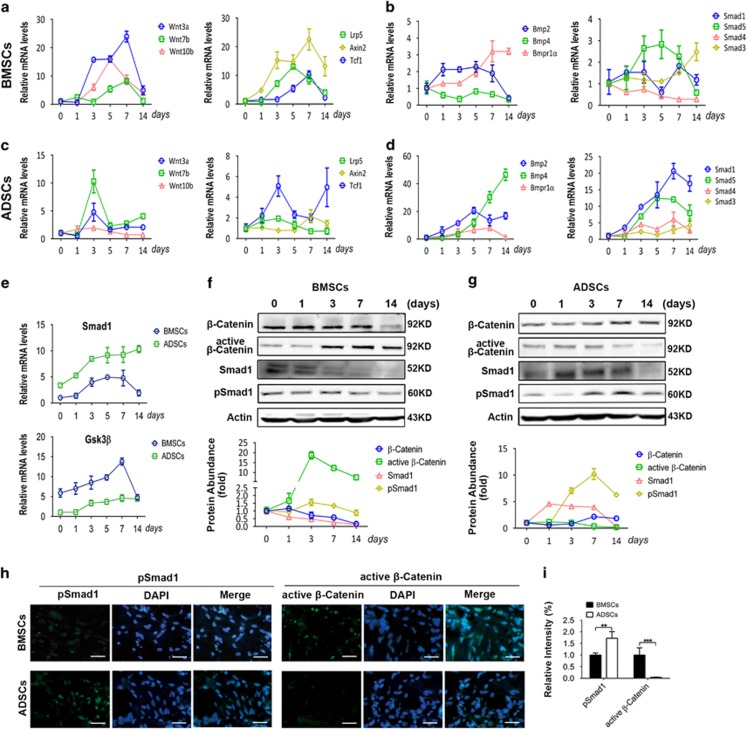
Wnt and BMP signaling are differentially activated during osteogenic differentiation of BMSCs and ADSCs. (**a–d**) Expression of Wnt pathway-related genes and BMP pathway genes at different time points of BMSC (**a** and **b**) and ADSC (**c** and **d**) osteogenic differentiation was determined by real-time RT-PCR. (**e**) *Smad1* and *GSK3β* mRNA expression in ADSCs and BMSCs during osteogenic differentiation was analyzed by real-time RT-PCR. (**f** and **g**) Western blot analysis of the markers of Wnt and BMP pathways during osteogenic differentiation of BMSCs (**f**) and ADSCs (**g**). *β*-Actin was used as the internal control. Relative protein abundance of each blots was normalized to the gray value of *β*-actin. (**h** and **i**) Immunofluorescence assay of p-Smad1 and active *β*-catenin in BMSC and ADSC transplants after 4 weeks of immunocompromised mice transplantation. The fluorescence intensity was quantified by Image Pro software (**i**). Scale bar: 200 *μ*m. Results are represented as mean±S.D. **P<0.01, ***P<0.001 (*n*=3)

We moved to the next question of why GSK3*β* and Smad1 have distinct importance in osteogenic differentiation of different MSCs. The effectivity of one signaling molecule is largely dependent on the activation status of the signaling pathway. Real-time RT-PCR showed that the expression of Wnt ligands was increased over 10-fold during the early stage of BMSC osteogenic differentiation, and the expression of Wnt receptor and downstream genes was also significantly increased till day 7 (Figure 5a). Comparatively, only a few BMP pathway genes were slightly increased during BMSC differentiation (Figure 5b). In contrast, the expression of BMP pathway ligands, receptor and downstream regulators was significantly enhanced during osteogenic differentiation of ADSCs (Figure 5d), and only parts of the Wnt pathway genes were modestly increased (Figure 5c). During osteogenic differentiation process, BMSCs expressed more *GSK3β* but less *Smad1* than ADSCs (Figure 5e).

Moreover, western blot analysis showed that active *β*-catenin protein accumulation, a marker of Wnt pathway activation, was increased >10-fold during the middle stage of osteogenic differentiation of BMSCs (Figure 5f). Conversely, phosphorylated Smad1 (pSmad1) protein accumulation, a marker of BMP pathway activation, was slightly decreased at day 7 of BMSC osteogenic differentiation (Figure 5f). On the contrary, pSmad1 protein level was significantly increased whereas active *β*-catenin protein accumulation was decreased during osteogenic differentiation of ADSCs (Figure 5g).

To confirm the signaling activation status *in vivo*, we performed immunofluorescence assay in MSCs transplants harvested after 4 weeks. Consistent with the *in vitro* results, active *β*-catenin protein level in BMSCs was significantly higher than that in ADSCs, whereas pSmad1 protein accumulation in BMSCs was lower than that in ADSCs (Figures 5h and i). Taken together, these results indicated that Wnt signaling is dominantly activated during osteogenic differentiation of BMSCs, whereas BMP signaling is more active during ADSC differentiation.

### BMSCs and ADSCs depend on different signaling pathways to promote osteogenic differentiation

**Figure 6:**
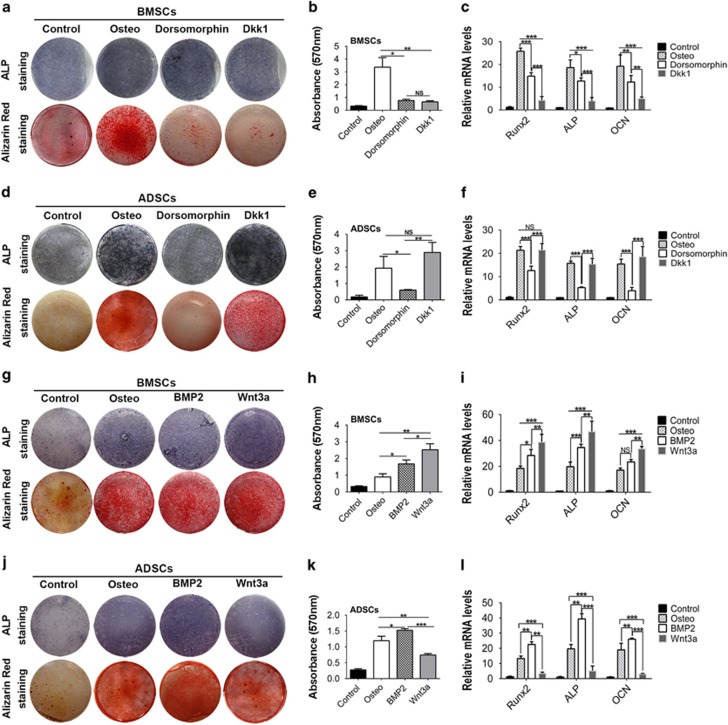
Wnt and BMP signaling pathways play different role in BMSC and ADSC osteogenic differentiation. (**a–c**) BMSCs were cultured in osteogenic medium with Dorsomorphin or DKK1. Then, ALP and alizarin red staining were performed separately after 7 and 14 days of induction (**a**). Alizarin red staining was quantified by spectrophotometer (**b**). Expression of *Runx2*, *Alp* and *Ocn* was measured by real-time RT-PCR (**c**). (**d–f**) ALP staining (**d**), alizarin red staining (**d** and **e**) and real-time RT-PCR analysis (**f**) in ADSCs cultured in osteogenic medium containing Dorsomorphin or DKK1. (**g–l**) ALP staining, alizarin red staining and real-time RT-PCR were performed in BMSCs (**g–i**) and ADSCs (**j–l**) cultured in osteogenic medium containing recombinant BMP2 or Wnt3a. Results are represented as mean±S.D. *P<0.05, **P<0.01, ***P<0.001 (*n*=3)

The differential activation of Wnt and BMP signaling suggested that BMSCs and ADSCs apply different signaling pathways to promote osteogenic differentiation. To certify the notion, we supplied Dorsomorphin to inhibit BMP pathway and Dickkopf-1 (DKK1) to inhibit Wnt pathway. Although both Dorsomorphin and DKK1 suppressed osteogenic differentiation of BMSCs, DKK1 was more effective than Dorsomorphin (Figures 6a–c). In ADSCs, Dorsomorphin significantly suppressed the osteogenic differentiation (Figures 6d–f). Interestingly, in contrast with its role in BMSC differentiation, DKK1 did not affect ADSC osteogenic differentiation (Figures 6d–f).

We next performed gain-of-function assay using recombinant Wnt3a to activate Wnt signaling and BMP2 to activate BMP signaling. Both BMP2 and Wnt3a enhanced the mineralized nodule formation and osteogenic markes expression in BMSCs, and Wnt3a was more effective than BMP2 (Figures 6g–i). In ADSCs, BMP2 markedly promoted the osteogenic differentiation of ADSCs, whereas Wnt3a slightly inhibited the osteogenic differentiation of ADSCs (Figures 6j–l). Taken together, these results indicated that Wnt pathway activation is indispensable for BMSC osteogenic differentiation, whereas BMP signaling activation is more important to promote ADSC osteogenic differentiation.

### MiR-26a regulates different signaling pathways in BMSCs and ADSCs

**Figure 7:**
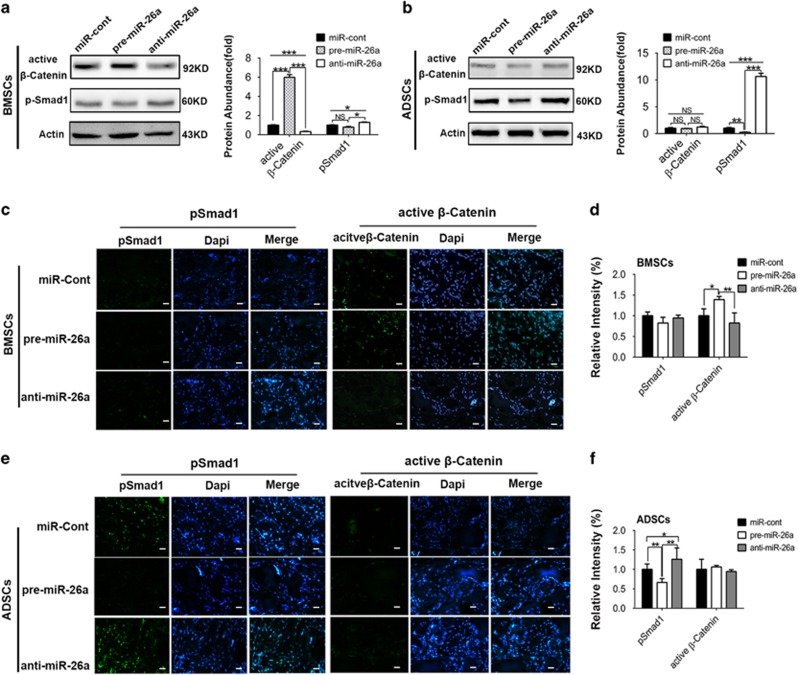
MiR-26a potently regulates different signaling pathways in BMSC and ADSC osteogenic differentiation. (**a** and **b**) BMSCs and ADSCs were transfected with negative control (miR-cont), miR-26a precursors (pre-miR-26a) and inhibitors (anti-miR-26a) for 48 h before osteogenic induction. Western blot was performed to analyze the protein level of active *β*-catenin or pSmad1 in BMSCs (**a**) and ADSCs (**b**) after 14 days of induction *in vitro*. Relative protein abundance of each blots was normalized to the gray value of *β*-actin. (**c–f**) BMSCs and ADSCs transfected with negative control (miR-cont), miR-26a precursors (pre-miR-26a) and inhibitors (anti-miR-26a) were implanted into nude mice for 4 weeks. Expression of pSmad1 and active *β*-catenin in transplants of BMSCs (**c** and **d**) and ADSCs (**e** and **f**) was detected by immunofluorescence assay, and quantified by Image Pro software. Scale bar: 200 *μ*m. Results represent means±S.D. *P<0.05, **P<0.01, ***P<0.001 (*n*=3)

Our results above showed that differential activation of Wnt and BMP pathways determined the distinct importance of GSK3*β* and Smad1 in BMSC and ADSC differentiation. To further verify the notion, we checked the effects of miR-26a on activation of Wnt and BMP pathways in different MSCs after 14 days of osteogenic induction. Overexpression of miR-26a increased active *β*-catenin expression, whereas knockdown of miR-26a decreased active *β*-catenin expression in BMSCs (Figure 7a). MiR-26a also inhibited pSmad1 protein slightly in BMSCs. As pSmad1 protein rarely expressed in BMSCs after osteogenic differentiation, the absolute quantity change of pSmad1 was much lower than that of active *β*-catenin (Figure 7a). Immunofluorescence assay of *in vivo* transplants revealed that miR-26a significantly inhibited the expression of GSK3*β* (Supplementary Figure S4A and B) and increased the level of active *β*-catenin (Figures 7c and d). As pSmad1 expression in BMSC implants was scarce, we did not detect the effect of miR-26a on pSmad1 level *in vivo* (Figures 7c and d).

In ADSCs, miR-26a markedly effected pSmad1 protein expression (Figure 7b). Although miR-26a slightly inhibited GSK3*β*, miR-26a knockdown or overexpression did not significantly affect the active *β*-catenin protein level in ADSCs. Immunofluorescence assay also confirmed that miR-26a inhibited the activation of Smad1 (Supplementary Figure S4C and D) and pSmad1 (Figures 7e and f) *in vivo*. We did not detect the regulation of miR-26a on active *β*-catenin of ADSC transplants (Figures 7e and f).

In conclusion, we found that BMP and Wnt signaling pathways were differentially activated and played distinct role in osteogenic differentiation of BMSCs and ADSCs. The different gene regulation program determined the opposite function of miR-26a in BMSC and ADSC osteogenic differentiation. Our findings uncovered that tissue-specific MSCs applied differential signaling regulation programs for cell-fate commitment, suggesting the complexity in tissue-specific stem cell research and application.

## Discussion

Both Wnt and BMP are critical pathways controlling MSC fate. Notably, WNT and BMP pathways are reported to be closely related with each other during osteogenic differentiation of MSCs. BMPs repress Wnt signaling in skeletal progenitor cells to control osteoblastic differentiation.^34, 35^ Studies using pluripotent mesenchymal cell lines show that BMP upregulates Wnt signaling to promote osteogenic differentiation.^36, 37^ Wnt3a, in turn, regulates BMP signaling in osteoblast.^38^ The interplay of BMP and Wnt signaling seems to be cell specific. In this study, we confirmed that Wnt and BMP signaling are antagonistically activated during osteogenic differentiation of BMSCs and ADSCs. Wnt ligand expressions were potently activated, but BMP expressions were slightly elevated during BMSC osteogenesis. In contrast, BMP expressions were enhanced much more potently than the expression of Wnt ligands during ADSC osteogenic differentiation, suggesting that only one signaling plays the dominate role in each cell. However, it remains elusive whether Wnt or BMP signaling directly inhibits each other in MSCs. Further gain- and loss-of-function studies are necessary to uncover the mechanism of BMP and Wnt signaling interplay in tissue-specific MSCs.

A number of comparative studies demonstrated a difference in the differentiation capacity of MSCs from specific tissues.^39, 40, 41, 42, 43^ However, there remains a question of whether tissue-specific MSCs apply different gene regulation programs for osteogenic differentiation. Some studies showed that many genes, including genes involving in important signaling pathways, are differentially expressed between different MSCs.^21, 22, 23, 24, 43^ Here, through study of the opposite function of miR-26a in BMSCs and ADSCs, we confirmed distinct signaling regulation networks between osteogenic differentiation of BMSCs and ADSCs. We thoroughly compared the activation states and function of Wnt and BMP pathways during BMSC and ADSC differentiation. Interestingly, we found that Wnt and BMP signaling were differentially activated during osteogenic differentiation of BMSCs and ADSCs. Function assay further confirmed that BMSCs majorly applied Wnt signaling to promote osteogenic differentiation, but ADSCs majorly used BMP signaling to facilitate osteogenic differentiation. In accordance with previous studies showing that WNT and BMP are differentially activated in MSCs derived from different tissues, our findings supported that tissue-specific MSCs apply distinct signaling pathways for differentiation. Further works are needed to uncover the physiological significance and the underlying mechanism of this phenomenon.

Notably, our results showed that Wnt signaling pathway potently promotes osteogenic differentiation of BMSCs, whereas it inhibits ADSC osteogenic differentiation, and this is supported by previous reports.^32, 44^ Very little is known about the molecular basis of this phenomenon. One possibility is that the tissue-specific epigenetic signatures determine transcription factors of Wnt and BMP pathways to activate different cohorts of genes. A deeper study of epigenetic signature between tissue-specific MSCs would be necessary to understand the gene regulation of signaling pathways.

MiR-26a regulates the differentiation of several cells, such as BMSCs, adipose progenitors, smooth muscle cells, monocytes and so on. MiR-26a targeted E2F7 to inhibit monocytic differentiation.^45^ MiR-26a was upregulated and targeted ten-eleven translocation (TET) enzymes during pancreatic cell differentiation.^46^ The transcription factors Smad1 and Smad4 were regulated by miR-26a to promote the differentiation of myoblasts.^47^ MiR-26 family and its downstream effector ADAM17 functioned in human adipocyte differentiation by promoting characteristics of energy-dissipating thermogenic adipocytes.^48^ These results suggested that miR-26a functions as an important regulator of cell lineage commitment. In our study, we identified miR-26a as a positive regulator of BMSC osteogenic differentiation but a negative regulator of ADSC osteogenic differentiation. Interestingly, it was reported that miR-26a is upregulated during adipogenesis of BMSCs,^9^ suggesting the more complicated role of miR-26a in MSCs osteogenic–adipogenic differentiation balance. As BMSCs are inclined to differentiate into osteoblasts and ADSCs prefer to differentiate into adipocytes in physiological conditions, miR-26a might be a crucial regulator to ensure normal lineage commitment of tissue-specific MSCs.

One microRNA might play different roles in different tissues. For example, miR-21 promoted the proliferation of squamous cell carcinoma and breast tumor cells,^49, 50^ but it inhibited the proliferation of ADSCs.^51^ MiR-214 promoted the differentiation of myocytes,^52^ whereas it inhibited the differentiation of osteoblasts.^53^ This could be explained as one miRNA potentially targets on a number of mRNAs. As gene expression varies in different types of cells, microRNA function is likely to be determined by which genes are highly expressed and functional. In our study, we find that Smad1 and GSK3*β* mRNA expression is quite different between BMSCs and ADSCs. Furthermore, Wnt signaling and BMP signaling is differentially activated and plays different roles to regulate osteogenic differentiation of MSCs. Thus, these differences might determine the opposing function of miR-26a in different MSCs. Therefore, further systemic research of signaling regulation program is necessary for understanding miRNA-mediated post-transcriptional regulation of tissue-specific MSCs.

## Materials and Methods

### Animals

C57BL/6J mice and BALB/c nude mice were obtained from the Laboratory Animal Research Centre of the Fourth Military Medical University (Xi'an, China). All procedures that involved animals were approved by the animal use and care committee of the Fourth Military Medical University (License Number: 2014 KQ-005). All mice were housed under specific pathogen-free conditions (22 °C, 12-h light/12-h dark cycle, and 50–55% humidity) with free access to food pellets and tap water.

### Materials

Recombinant mouse BMP2 (R&D, Minneapolis, MN, USA), recombinant mouse Wnt3a (R&D), recombinant mouse Dkk1 (PeproTech, Rocky Hill, NJ, USA) and dorsomorphin (Santa Cruz Biotechnology, Santa Cruz, TX, USA) were obtained commercially.

### Cell culture and identification

The 4- to 6-week-old C57BL/6J mice were used for cell culture. BMSCs were isolated and cultured as previously described.^54^ Mouse were killed and the hindlimbs were aseptically removed and bones were dissected free of soft tissues. Marrow cavities of femur and tibia were flushed with culture medium as *α*-MEM (Invitrogen, Carlsbad, CA, USA) supplemented with 10% FBS, 1% penicillin and streptomycin and 2-mercaptoethanol (2-ME). 1.5 × 10^7^ cells were seeded in 10 cm tissue culture flasks and incubated in humidified atmosphere of 5% CO_2_ at 37 °C. Nonadherent cells were removed by frequent medium change during 72 h. The remaining adherent colonies were cultured for 14 days until confluent, and passaged after digestion with 0.25% trypsin for 3 min.

ADSCs were isolated from scraps of subcutaneous adipose tissues as previously described.^55^ In brief, the adipose tissues were minced and incubated in 1% collagenase type I for 1.5 h in a 37 °C water bath shaker. The digested solution was filtered through a 200-*μ*M and a 100-*μ*m cell strainer to separate undigested tissue fragments. Adipocytes and aqueous supernatant were separated by centrifugation at 800 × *g* for 5 min. Then, the deposit was resuspended in culture medium and plated in 10 cm tissue culture flasks and maintained in humid incubator at 37 °C and 5% CO_2_. Hematopoietic lineage cells attached to Petri dish after 1 h were removed, and the nonadherent cells containing ADSC populations were fed with *α*-MEM supplemented with 10% FBS, 1% penicillin and streptomycin until colony forming and reaching 70–80% confluence.

Cells at passage 3 were used for all experiments. Both BMSCs and ADSCs highly expressed MSC markers (Sca-1, CD90, CD106) and did not express hematopoietic cell marker (CD34), and had self-renewal and multipotent differentiation potential (Supplementary Figures S1A–K).

### Osteogenic differentiation and mineralization assay

After the cells reached 70–80% confluence, differentiation medium containing *α*-MEM, 10% FBS, 100 *μ*g/ml ascorbic acid, 2 mM *β*-glycerophosphate and 10 nM dexamethasone was used to induce osteogenic differentiation of both MSCs. The medium was changed every 3 days. After induction for 7 days, ALP staining was performed with the BCIP/NBT Alkaline Phosphatase Color Development Kit (Beyotime Co., Shanghai, China) following the standard protocol. After 14 days of osteogenic differentiation, 1% alizarin red (Sigma, St. Louis, MO, USA) was used to detect calcium accumulation according to the manufacturer's suggested protocol. Then, alizarin red was incubated with 100 mM cetylpyridinium chloride (Sigma) for 30 min at room temperature and quantified by spectrophotometer at 540 nm. The final calcium levels in each group were normalized with the total protein concentration obtained from the duplicate plates.

### Real-time RT-PCR of mRNA and miRNA

Total RNA was isolated using Trizol reagent (Invitrogen) according to the manufacturer's standard instructions. For reverse transcription of mRNA, random-primed cDNA was synthesized from 2 *μ*g of total RNA using a PrimeScript RT Reagent Kit (TaKaRa, Dalian, China). Real-time RT-PCR analysis was performed using the SYBR Premix Ex Taq II Kit (TaKaRa) and detected on the ABI Prism 7500 HT Sequence Detection System (Applied Bio-Systems, Foster City, CA, USA). *β*-Actin and U6 were used as loading controls for quantitation of mRNA and microRNA. The optimized microRNA-specific primers for miR-26a and the endogenous control U6 were commercially obtained (RiboBio, Guangzhou, China). Primers of PCR are listed in Supplementary Table S1.

### Western blot analysis

Cells were harvested in RIPA lysis buffer (Beyotime Co.). Whole-cell protein extracts were quantified by the BCA assay, separated on 12% SDS-PAGE and then transferred to PVDF membranes (Millipore, Billerica, MA, USA) and blocked with 5% nonfat milk powder in PBST for 3 h. The membranes were probed overnight with the primary antibodies and then 4 h with peroxidase-conjugated secondary antibodies (Boster, Wuhan, China). Antibodies used in this study included the mouse *β*-Actin, rabbit Smad1, rabbit phospho-Smad1, mouse GSK3*β*, rabbit phospho-GSK3*β* (Cell Signaling, Beverly, MA, USA), rabbit *β*-catenin (Abcam, Cambridge, MA, USA) and mouse active *β*-catenin (Millipore). The blots were visualized using an ECL Kit (Amersham Biosciences, Piscataway, NJ, USA) according to the manufacturer's recommended instructions. To measure the protein abundance, gray value of the blots in scanned images was measured with ImageJ Plus software (National Institutes of Health, Bethesda, MD, USA). Gray value of each target protein was normalized to that of *β*-Actin before comparison.

### The *in vivo* bone formation assay

BALB/c nude mice, 6 weeks old, were used as hosts for implantation. BMSCs and ADSCs were first transfected with miR-26a precursors, inhibitors or negative controls as described before, and then cultured in osteogenic differentiation medium for 3 days. Approximately 4 × 10^6^ cells were mixed with 40 mg hydroxyaptite-tricalcium phosphate (HA-TCP) powders (Sigma-Aldrich, St. Louis, MO, USA) and subcutaneously implanted into the dorsal surface of immunocompromised mice. Implants were harvested after 4 or 8 weeks for immunofluorescence and histological assay separately.

### H&E staining of MSC implants

MSC implants separated from surrounding fibrous capsule were fixed in 4% neutral buffered formalin (Sigma) overnight and decalcified with EDTA (10%) (Sigma) for 2 weeks. Then, the implants were embedded in paraffin and sectioned at 5 mm. H&E staining was performed according to standard protocols. Photos were taken under magnification of × 40 on four consecutive microscopic fields of the sections of implants. Osteoid formation in transplants was semiquantified as osteoid area per total area in H&E staining photos with Image Pro software (Media Cybernetics, Silver Spring, MD, USA).

### Immunofluorescence assay of MSC implants

Decalcified MSC implants were dehydrated in 30% sucrose solutions until submerged in bottom. Samples were embedded into optimal cutting temperature compound (OCT) for frozen sections, and analyzed by immunofluorescence assay following the standard protocol. Antibodies used in this study including goat Smad1 (Santa Cruz Biotechnology), mouse active *β*-catenin (Millipore), rabbit phospho-Smad1 and rabbit GSK3*β* (Cell Signaling). The samples were treated with fluorescence-labeled secondary antibodies and the nuclei were stained with 100 ng/ml of 4',6-diamidno-2-phenylinde (DAPI) (Beyotime Co.). All samples were examined under a confocal microscope (Olympus Optical, Tokyo, Japan).

### Transfection of miRNA precursors and inhibitors

Cells were seeded into 12-well or 6-well plates and transfection was performed shortly before or at 70% confluence. The microRNA precursors, inhibitors and negative controls of miR-26a were commercially purchased (Ambion, Austin, TX, USA). The cells were transfected with pre-miR-26a and negative control at a final concentration of 50 nM, and anti-miR-26a of 100 nM. Cell suspensions were overlaid onto the transfection complexes and incubated at 37 °C and 5% CO_2_ for 24 h until further study.

### Transfection of siRNA

Cells were seeded into 12-well or 6-well plates and transfected at 40–60% confluence. The Smad1 siRNA sequence (5′-GAACUGAAGCCUCUGGAAU-3′, 3′-CUUGACUUCGGAGACCUUA-5′), Gsk3*β* siRNA sequence (5′-GGUAUAUCAAGCCAAACUU-3′, 3′-CCAUAUAGUUCGGUUUGAA-5′) and scramble RNA were commercially synthesized (RiboBio). The cells were transfected with miRNAs together with Smad1 siRNA or Gsk3*β* siRNA by Lipofectamine 2000 (Invitrogen) according to the manufacturer's instructions, and transected with scramble RNA as a negative control at the same time. Final concentration of microRNA precursors, negative control and siRNA were 50 nM, and the concentration of inhibitor was 100 nM. The cells were incubated at 37 °C in the incubator for 24 h before further study.

### Luciferase reporter assay

To construct a vector of mouse Smad1 and GSK3*β*, the Smad1 oligonucleotide sequence were amplified using primers (forward: 5′- GGCTCCTTCGTCAGGTCTCCA-3′ reverse: 5′-ACGGATGAAATAGGATTGTGGG-3′) with *Hin*dlll and *Spe*l sites at their extremities to insert the pMIR-Report vector (Ambion). GSK3*β* oligonucleotide sequences were amplified using primers (forward: 5′- CCACCATCCTTATCCCTCCAC-3′ reverse: 5′-GGAGGAGCAGAGCATTAAACACA-3′). HEK293T cells were seeded in a 96-well plate at 50 to 60% confluence. After 24 h, cells were co-transfected with reporter constructs as pMIR-Report (pMIR-Cont), pMIR-Smad1 or pMIR-GSK3*β* plasmids (125ng), and microRNA controls, precursors or inhibitors (20 pmol). All transfections were conducted using Lipofectamine 2000 (Invitrogen). Luciferase assays were performed 48 h after transfection using the Dual Luciferase Reporter Assay System (Promega, Madison, WI, USA). Firefly luciferase activity was normalized to *Renilla* luciferase activity for each transfected well. Each transfected well was assayed in triplicate.

### Statistical analysis

Data are presented as mean±S.D. Comparisons were analyzed using Student's two-tailed *t*-test or one-way ANOVA for experiments with more than three groups. All experiments were repeated at least three times, and representative experiments are shown. Differences were considered to be significant when *P*<0.05.

## Abbreviations

ADSC: adipose tissue-derived mesenchymal stem cell
ALP: alkaline phosphatase
BMP: bone morphogenetic protein
BMSC: bone marrow-derived mesenchymal stem cell
DKK1: Dickkopf-1
HA-TCP: hydroxyaptite-tricalcium phosphate
miRNA: microRNA
MSC: mesenchymal stem cell
OCN: osteocalcin
pSmad1: phosphorylated Smad1
RT-PCR: reverse transcription PCR
WNT: wingless-type MMTV integration site family
