# Supplementary material for: MiR-26a functions oppositely in osteogenic differentiation of BMSCs and ADSCs depending on distinct activation and roles of Wnt and BMP signaling pathway
Source: Cell Death Dis. 2015 Aug 6;6(8):e1851–. doi: 10.1038/cddis.2015.221 (PMC4558512; doi:10.1038/cddis.2015.221)
Supplement: Supplementary Information [file cddis2015221x1.doc]

**Summary of Supplementary Files content**

- Supplementary_information.doc: Contains Supplementary Figure Legends.
- Supplementary Table S1.doc: Supplementary Table S1.
- Supplementary Figure S1.jpg: Supplementary Figure S1.
- Supplementary Figure S2.jpg: Supplementary Figure S2.
- Supplementary Figure S3.jpg: Supplementary Figure S3.
- Supplementary Figure S4.jpg: Supplementary Figure S4.
