# Supplementary material for: MiR-26a functions oppositely in osteogenic differentiation of BMSCs and ADSCs depending on distinct activation and roles of Wnt and BMP signaling pathway
Source: Cell Death Dis. 2015 Aug 6;6(8):e1851–. doi: 10.1038/cddis.2015.221 (PMC4558512; doi:10.1038/cddis.2015.221)
Supplement: Supplementary Table S1 [file cddis2015221x2.doc]

### Supplementary Table 1. Primers for Realtime RT-PCR

| Primer name | Sequence (5’ to 3’) |
| --- | --- |
| *β-actin* | F: 5’ CTGGCACCACACCTTCTACA 3’  R: 5’ GGTACGACCAGAGGCATACA 3’ |
| *Runx2* | F: 5’ CCGCACGACAACCGCACCAT 3’  R: 5’ CGCTCCGGCCCACAATCTC 3’ |
| *Ocn* | F: 5’ AAGCAGCAACGCTAGAAGACAG 3’  R: 5’ GCGCCGGAGTCTGTTCACTA 3’ |
| *Alp* | F: 5’ TTGTGCCAGAGAAAGAGA 3’  R: 5’ GTTTCAGGGCATTTTTCAAGG 3’ |
| *Bmp2* | F: 5’ ACGGACTGCGGTCTCCTAAAG 3’  R: 5’ TTGCTGGGGGTGGGTCTC 3’ |
| *Bmp4* | F: 5’ CCGAGCCAACACTGTGAGG3’  R: 5’ GGTGAAGAGGAAACGAAAAGCA3’ |
| *Bmpr1a* | F: 5’ GGGGTCGTTACAACCGTGAT 3’  R: 5’ CTGTTTGGCAATAGTTCGCTGA 3’ |
| *Smad1* | F: 5’ CACCTGCTTACCTGCCTCCT 3’  R: 5’ CAACTGCCTGAACATCTCCTCTG 3’ |
| *Smad5* | F: 5’ AAGAATGAGGCTGTTGGTGTAAG 3’  R: 5’ AGAAAGAGGCAGAGCGTTGAT 3’ |
| *Smad4* | F: 5’ ACCATCATAACAGCACTACCACCT 3’  R: 5’ CATAGCCATCCACAGTCACAACA 3’ |
| *Smad3* | F: 5’ AGGGGCTCCCTCACGTTATC 3’  R: 5’ CATGGCCCGTAATTCATGGTG 3’ |
| *Wnt3a* | F: 5’ TGCTGTTGAGGCAATGGTC3’  R: 5’ CAGATGGGCTGTATGTA 3’ |
| *Wnt7b* | F: 5’ GGAGAAGCAAGGCTACTACAACC 3’  R: 5’ ACCACAAAGCGACGAGAAAAG 3’ |
| *Wnt10b* | F: 5’ TTCTTGGCTTTGTTCAGTCGG 3’  R: 5’ ACTCGTGAACGGCGATGTG 3’ |
| *Lrp5* | F: 5’ ACGTCCCGTAAGGTTCTCTTC 3’  R: 5’ GCCAGTAAATGTCGGAGTCTAC 3’ |
| *Axin2* | F: 5’ GACGCACTGACCGACGATT 3’  R: 5’ GAAGGCAGCAGGTTCCACA 3’ |
| *Tcf1* | F: 5’ CCTGACCGAGTTGCCTAATGG 3’  R: 5’ ATGGGTCCTCCTGAAGAAGTGA 3 |
| *Gsk3β* | F: 5’ CAAGCAGACACTCCCTGTGAT 3’  R: 5 ATGAAACATTGGGCTCTCCTC 3’ |
