# Supplementary material for: MiR-26a functions oppositely in osteogenic differentiation of BMSCs and ADSCs depending on distinct activation and roles of Wnt and BMP signaling pathway
Source: Cell Death Dis. 2015 Aug 6;6(8):e1851–. doi: 10.1038/cddis.2015.221 (PMC4558512; doi:10.1038/cddis.2015.221)
Supplement: Supplementary Figure Legends [file cddis2015221x3.doc]

## Supplementary Figure Legends

**Supplementary Figure S1**. Characterization of BMSCs and ADSCs. **(A):** The immuno-phenotype of BMSCs and ADSCs was examined by flow cytometry. **(B):** 1×10^4^ BMSCs or ADSCs were transplanted into 24 well plates. The cell number was counted every day to measure the proliferation. **(C):** One thousand BMSCs or ADSCs were transplanted in 10cm culture dish for 3 weeks. Toluidine blue staining was performed to show the colonies formed by MSCs. **(D, F):** ALP protein activity in BMSCs **(D, upper)** and ADSCs **(F, upper)** before and after 7-day of osteogenic induction was determined by ALP staining. Mineralized nodule formation of BMSCs **(D, below)** and ADSCs **(F, below)** before and after 14-day of induction was determined by alizarin red staining. **(E, G):** Expression of *Alp* and *Ocn* in BMSCs **(E)** and ADSCs **(G)** after induction was determined by realtime RT-PCR. **(H, J):** Oil red O staining was operated to detect the lipid accumulation in BMSCs **(H)** and ADSCs **(J)** cultured with adipogenic induction medium for 7 days. **(I, K):** Realtime RT-PCR was performed to analyze the expression of *PPARγ* and *LPL* in BMSCs **(I)** and ADSCs **(K)**. Data represent means ± SD. **P<0.01, ***P<0.001, n=3.

**Supplementary Figure S2**. Transfection efficiency of miR-26a precursors and inhibitors. MiR-26a precursors (pre-miR-26a), inhibitors (anti-miR-26a) and negative control (miR-cont) were transfected into BMSCs and ADSCs. Realtime RT-PCR was performed to detect miR-26a expression after 48h or 14 days. Data represent means ± SD.*P<0.05, **P<0.01, ***P<0.001, n=3.

**Supplementary Figure S3**. The efficiency of siRNA of Smad1 and GSk3β. Smad1 siRNA, GSK3β siRNA and negative control were transfected into BMSCs and ADSCs. Realtime RT-PCR analysis of target genes *Smad1* and *GSK3β* was performed 48h later. Data represent means ± SD. ***P<0.001, n=3.

**Supplementary Figure S4**. Expression of GSK3β and Smad1 in BMSCs and ADSCs in vivo. Subcutaneous implants of BMSCs and ADSC transfected with negative control (miR-cont), miR-26a precursors (pre-miR-26a) and inhibitors (anti-miR-26a) were harvested after 4 weeks. Expression of GSK3β and Smad1 in transplants of BMSCs (A, B) and ADSCs (C, D) were determined by immunofluorescence assay, and quantified by Image Pro software. Scale bar: 200μm. Data represent means ± SD. *P<0.05, **P<0.01, n=3.
