# Supplementary figures and images for: MiR-26a functions oppositely in osteogenic differentiation of BMSCs and ADSCs depending on distinct activation and roles of Wnt and BMP signaling pathway
Source: Cell Death Dis. 2015 Aug 6;6(8):e1851–. doi: 10.1038/cddis.2015.221 (PMC4558512; doi:10.1038/cddis.2015.221)

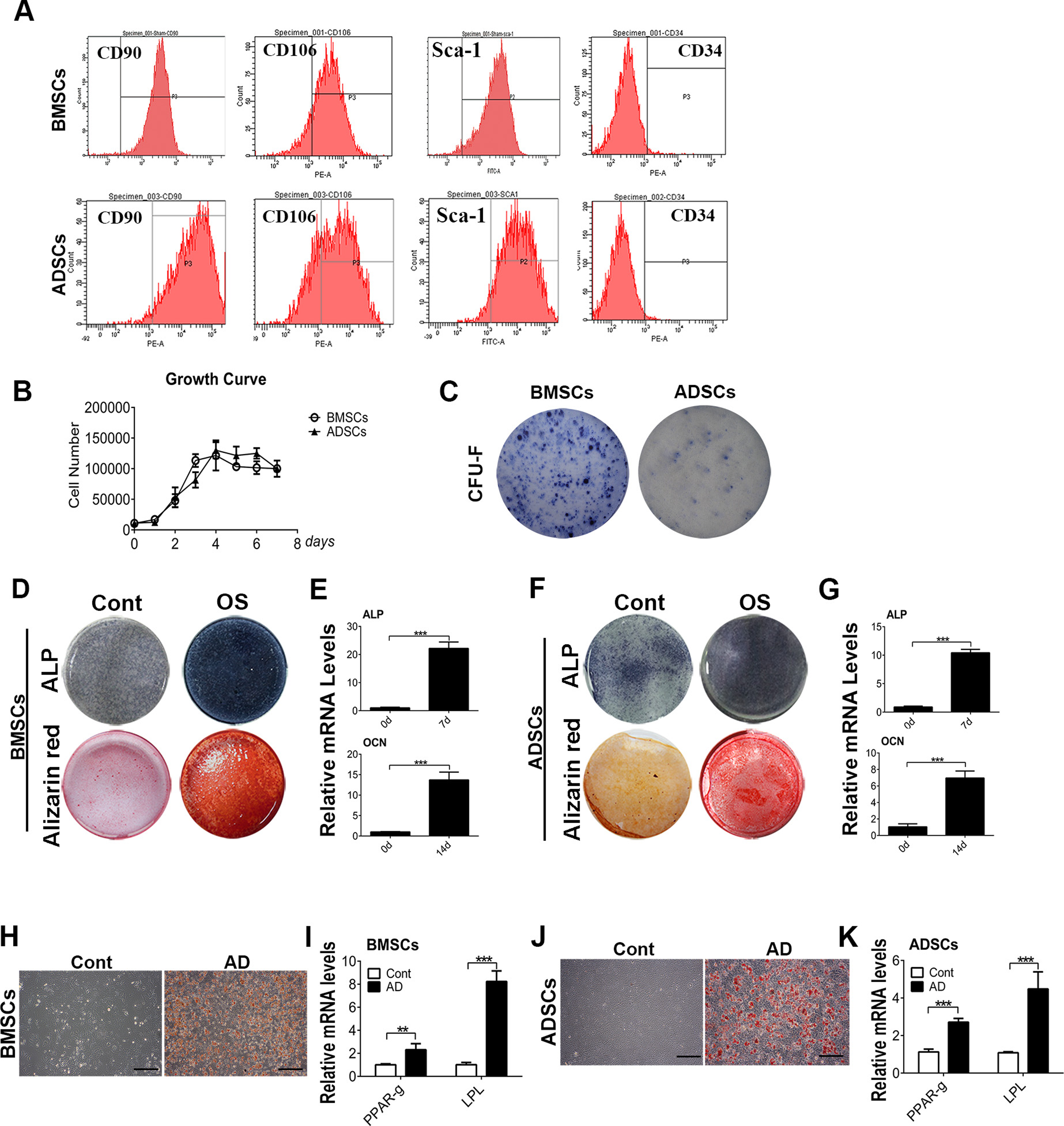

Supplement: Supplementary Figure S1 [file cddis2015221x4.tif]

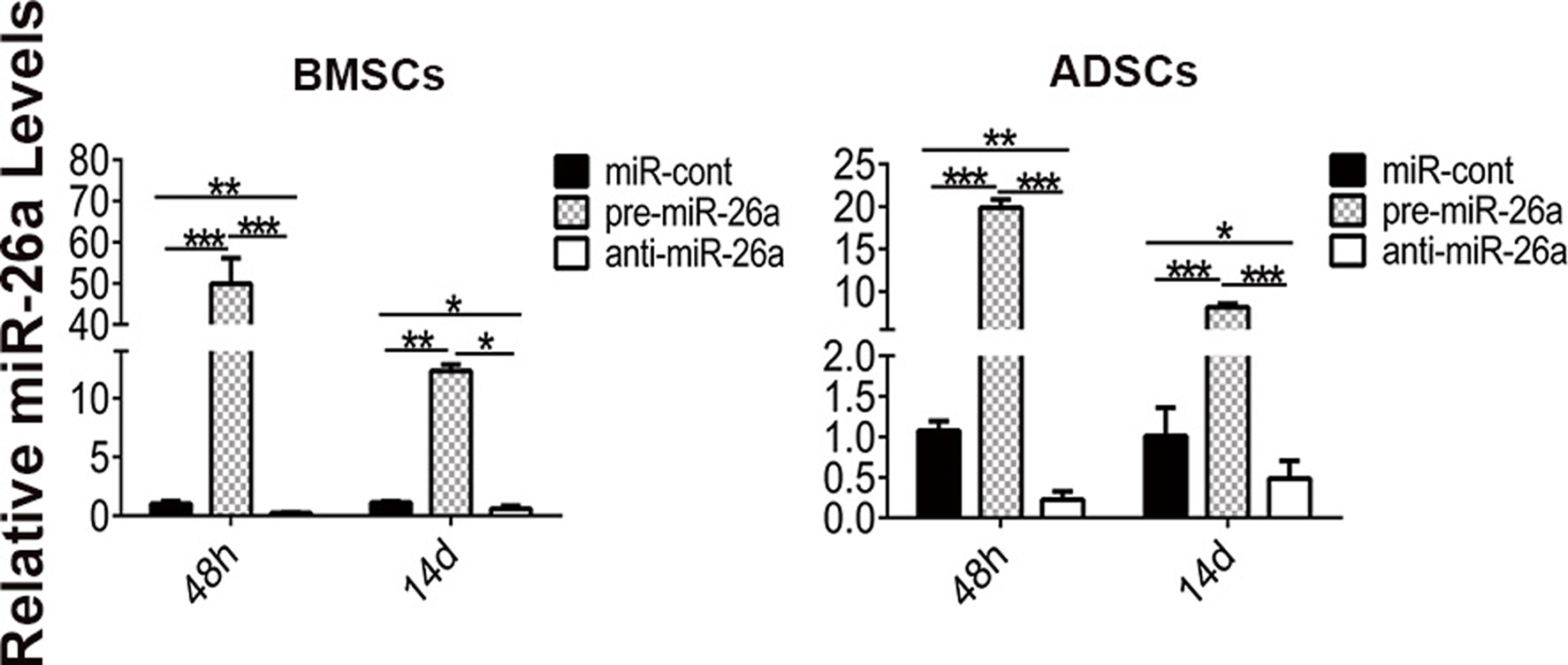

Supplement: Supplementary Figure S2 [file cddis2015221x5.tif]

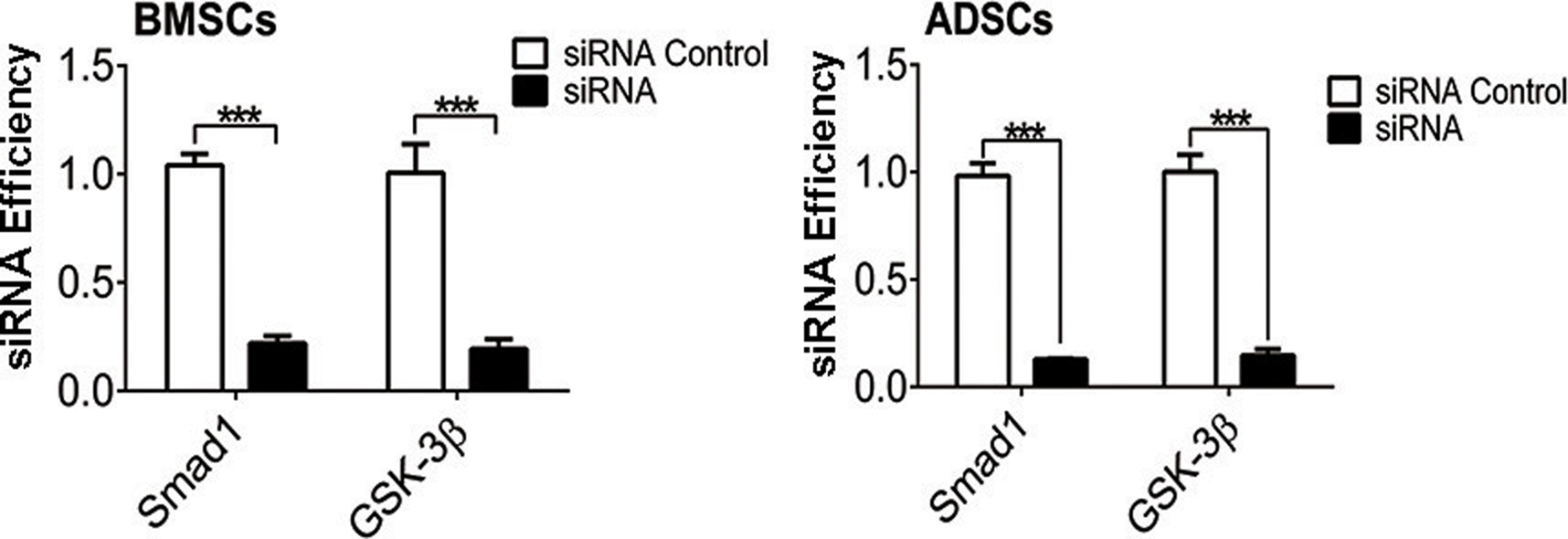

Supplement: Supplementary Figure S3 [file cddis2015221x6.tif]

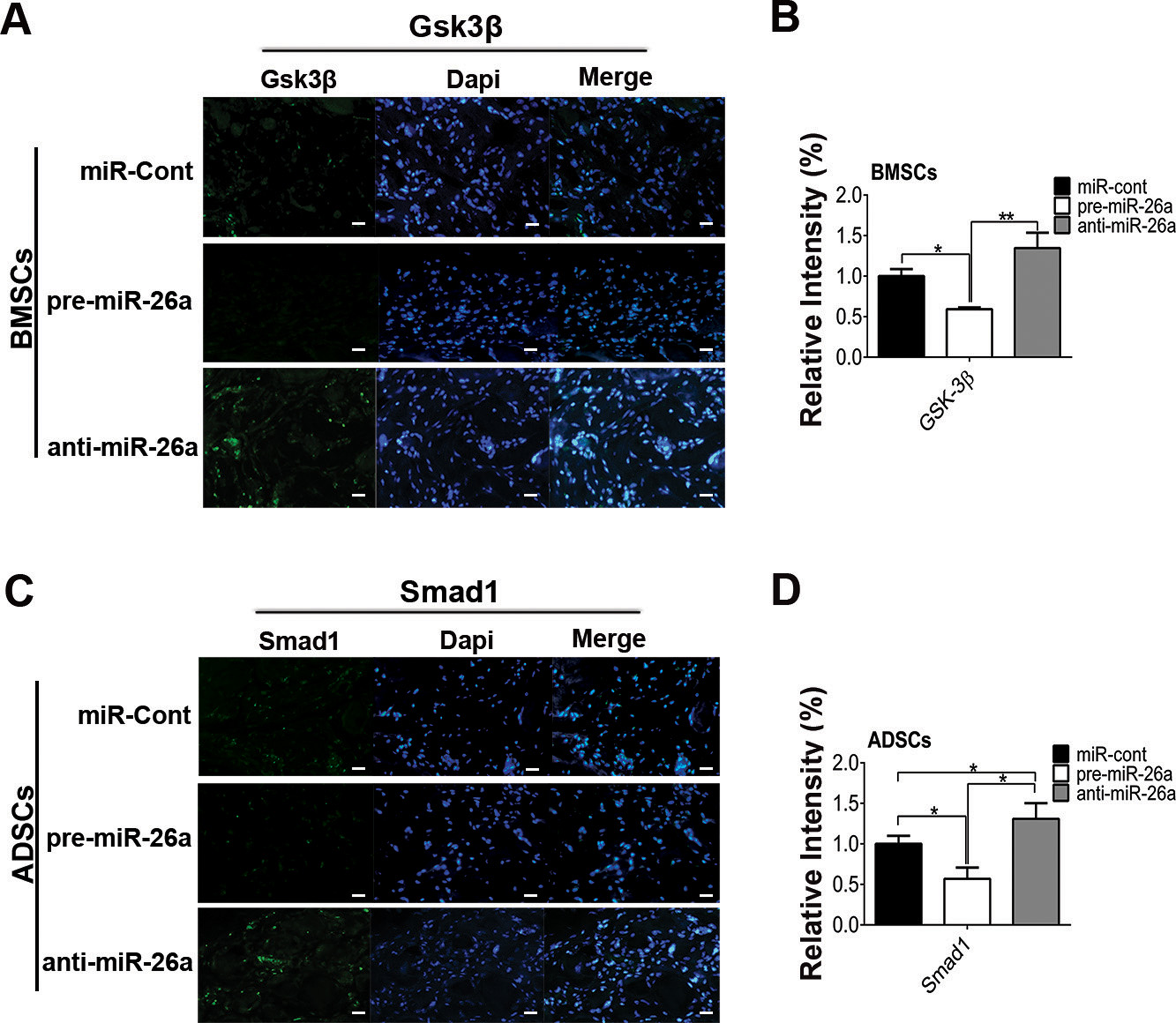

Supplement: Supplementary Figure S4 [file cddis2015221x7.tif]
